# Pandemic Legion History More Complex than Previously Thought

**DOI:** 10.1128/mBio.02377-20

**Published:** 2020-10-09

**Authors:** Jean-Nicolas Tournier

**Affiliations:** a Institut de Recherche Biomédicale des Armées (IRBA), Microbiology and Infectious Diseases Department, Brétigny sur Orge, France; b Institut Pasteur, Innovative Vaccine Laboratory, Paris, France; c Ecole du Val-de-Grâce, Paris, France; Albert Einstein College of Medicine

**Keywords:** emerging infectious diseases, tuberculosis, *Mycobacterium tuberculosis*, *Helicobacter pylori*, measles, smallpox, variola virus

## LETTER

I read with much interest the Perspective article by Morens et al. entitled “Pandemic COVID-19 Joins History’s Pandemic Legion” (1).

Morens and colleagues describe in the “early pandemic history” section the story of pathogens emerging around 12,000 years ago at the time of Neolithic agricultural revolution. As such, diseases such as “measles, smallpox, tuberculosis (TB), [and] gastric cancer (caused by Helicobacter pylori)” are cited as consequences of “conditions of intense human-animal proximity and environmental alterations.” This assertion of the dating of the origin of these aforementioned pathogens is partially misleading. Both viruses (i.e., those causing measles and smallpox) emerged probably much later, while Mycobacterium tuberculosis and Helicobacter pylori started their association with humans before the agricultural revolution.

Historical records of viruses are scarce, and the reconstruction of their evolutionary history might be difficult (2–4). However, a recent phylogenetic study of a 1912 strain has placed measles virus (MV) divergence from rinderpest virus during the sixth century before the Common Era (BCE), possibly coinciding with the rise of large cities allowing measles epidemic sustainability (5).

For smallpox, the exact date of divergence of variola virus (VARV) from a zoonotic strain is more disputed, as molecular data gave an estimation of emergence for the most recent common ancestor between the 16th and the 17th century, while skin lesions seen in the mummy of Ramses V, who died in 1157 BCE, suggested earlier interactions (6). Camelpox virus and taterapox virus infecting gerbils very likely shared a common ancestor with VARV that might have evolved from a common rodent orthopoxvirus (7, 8). A recent study of ancient VARV samples from Viking remains discovered a sister clade and predated VARV emergence as early as 603 CE, confirming written accounts of likely smallpox infections in Europe from the late 6th century (6).

In contrast, the interactions of M. tuberculosis and H. pylori with humans represent much more sophisticated and longer stories. A thorough phylogeographic analysis of modern H. pylori diversity has shown that the *Homo sapiens* became its specific host before he started his migration out of Africa 60,000 years ago and no later than 100,000 years ago (14). For TB, recent studies have clearly demonstrated that in opposition to a frequently reported idea, human TB is not a zoonosis derived from bovine TB arisen as a consequence of cattle domestication (9). M. tuberculosis and Mycobacterium bovis share more than 99.95% nucleotide identity, and while they probably do have a common ancestor, M. bovis is definitely not the parent of M. tuberculosis (10, 11). Concerning M. tuberculosis emergence dating, several studies are conflicting (11), but at least one study estimated that the most recent common ancestor of M. tuberculosis existed 70,000 years ago (12).

Moreover, on Yersinia pestis, the agent of plague, Morens and colleagues mentioned the “plague of Athens” (430 to 425 BCE) as “perhaps the first recorded pandemic,” although there is a wealth of data proving a much earlier presence of Y. pestis starting at least 4,900 years ago (4, 13).

Eventually, the conception of the agricultural revolution affecting health through direct emerging infections from domesticated livestock needs to become more balanced. The MV example has shown that emergence by accident is not the rule and that a pathogen needs more conditions than a close contact with human for sustainable success.
